# Using Polars to Improve String Similarity Performance in Python

**DOI:** 10.23889/ijpds.v9i5.2801

**Published:** 2024-09-18

**Authors:** Jeremy Foxcroft, Luiza Antonie

**Affiliations:** 1 University of Guelph

## Objective

String similarity is central to textual record linkage, and is often calculated with Levenshtein distance or Jaro-Winkler distance. In polars-strsim, we leverage the Polars DataFrame interface to surpass all existing Python libraries in computing these distances. Polars-strsim is especially useful for larger-than-memory datasets.

## Objectives and Approach

Polars is a library built to analyse and manipulate tabular data. It is written in Rust, but can be called from Python for ease of use. Polars-strsim implements the Levenshtein and Jaro-Winkler algorithms as a Polars extension.

We compare performance against nine other Python libraries on three million pairs of first names.

## Results

When computing Levenshtein/Jaro-Winkler distance for this benchmark, polars-strsim is respectively 5x/4x faster than the current fastest Python alternative and 47x/35x faster than the median.

The Polars streaming engine can be seamlessly used to manipulate larger than memory datasets. One practical application of this in record linkage is to find all pairs of records in a dataset where the Levenshtein distance computed across one (or more) fields is below a certain threshold (e.g., during blocking). With polars-strsim, this calculation can be performed in Python with CPU parallelism even if the input and/or output doesn’t fit in memory.

## Conclusions/Implications

This work introduces a powerful library for record linkage practitioners. Polars-strsim provides the convenience of Python, the performance of a strongly/statically typed compiled language, and the full power of the Polars streaming engine when computing string similarity, and can be updated to implement additional similarity algorithms.

